# Computed tomography identifies the proximodorsomedial subchondral bone of equine central tarsal bones as a predilection site for sclerosis, demineralisation and associated fractures

**DOI:** 10.1111/evj.70001

**Published:** 2025-07-24

**Authors:** Sandra Campana, Marie Dittmann, Patrick Kircher, Brice Donati

**Affiliations:** ^1^ Clinic for Diagnostic Imaging Vetsuisse Faculty, University of Zurich Zürich Switzerland; ^2^ Department of Livestock Sciences Research Institute of Organic Agriculture FiBL Frick Switzerland

**Keywords:** central tarsal bone, computer tomography, fracture, horse, sclerosis, stress remodeling

## Abstract

**Background:**

The distribution pattern of central tarsal bone (CTB) changes has not been described, except for slab‐ and dorsomedial–plantarolateral fractures.

**Objectives:**

To describe CTB changes in CT and document their distribution and associations.

**Study Design:**

Retrospective case series.

**Methods:**

Standing and recumbent tarsal CT studies from 94 clinical cases were retrospectively evaluated. General case information, degree of sclerosis (none‐severe), lesions (demineralisation, cystoid, fissure/fracture) and their location were recorded, dividing CTBs into 8 regions.

**Results:**

Eighty five of 94 tarsi showed at least one region of moderate to severe sclerosis, of which 90% affected the dorsomedial region. The prevalence of lesions was significantly associated with higher degrees of sclerosis (*p* = 0.04) at this site. Of 32 demineralising lesions, 21 were in the proximal subchondral bone dorsomedially. Twenty‐four CTBs showed fissures/fractures; 19/24 were in a dorsomedial–plantarolateral direction, and 17/19 were associated with demineralisation. Of five fissures/fractures with different configurations, none had associated demineralisation. There were 27 cyst‐like lesions, 21/27 in the distal subchondral bone, of which almost half (13/27) located medially.

**Main Limitations:**

Retrospective design; heterogeneous, warmblood‐oriented population; no clinical correlation of findings nor histological confirmation of described changes.

**Conclusions:**

Given the links between sclerosis, demineralisation and fissures/fractures, the dorsomedial proximal subchondral bone plate of the CTB must be scrutinised both in CT and radiography.

## 1 INTRODUCTION

Distal tarsal pathology is common in horses, and arthrosis of the distal tarsal joints (aka bone spavin) is historically the most commonly recognised entity.^1^, ^2^, ^3^ The central tarsal bone (CTB) is frequently affected as it contributes with subchondral bone to both the proximal and distal intertarsal joints. Changes secondary to arthrosis include osteophytosis, subchondral sclerosis and subchondral osteolysis. Among changes independent of arthrosis, fractures have been relatively commonly described. Sclerosis, demineralisation or osteolysis have been more rarely reported and are often interpreted as stress remodelling.^4^, ^5^, ^6^, ^7^, ^8^, ^9^

History and clinical signs in horses with CTB fractures can be unspecific and include regional swelling, effusion of the tarsocrural joint and positive distal tarsal blocks.^2^, ^10^ Peroneal and tibial nerve blocks and tarsometatarsal or tarsocrural joint anaesthesia may abolish or improve associated lameness.^7^

In racehorses, CTB fractures most commonly show slab configuration in a dorsal or slightly oblique plane.^11^ A conformational predisposition has been identified and loading of incompletely ossified small tarsal bones in foals can result in abnormally wedge‐shaped third tarsal bones and CTBs, which are then associated with slab fractures.^12^ In radiography, dorsal slab fractures are best appreciated in lateromedial projections depending on their conformation.^8^, ^11^, ^13^

More recently, after isolated reports,^9^, ^14^ two separate publications almost simultaneously described a consistently dorsomedial–plantarolateral (DMPL) configuration for CTB fractures in non‐racehorses.^7^, ^8^ These were more readily detected in slightly DMPL‐oblique radiographic projections or with computed tomography (CT), often showing concurrent sclerosis and occasionally ill‐defined fracture margins, both attributed to chronicity. Two other studies confirmed a consistent DMPL orientation of CTB fractures in their populations. The first reported a common coincidence of DMPL fractures with sclerosis,^6^ but postulated that some degree of increased bone density represented normal adaptation of the CTB to stress, in particular in sport horses. The second study reported incomplete fractures of the CTB in DMPL orientation, only affecting the proximal dorsomedial aspect of the CTB. Besides marked sclerosis, this study also described variable degrees of bone marrow lesion surrounding the fractures and osteoarthropathy of the adjacent joints.^5^ A stress‐related aetiology was proposed, highlighting similarities with other more commonly reported areas affected by incomplete stress fractures, such as the sagittal groove of the proximal phalanx.^5^

Independently of their orientation, radiographic detection of CTB fractures can be difficult depending on the degree of displacement and chronicity, and multiple projections can be advantageous in identifying them.^10^, ^15^ Scintigraphy can help diagnose CTB fractures, especially in the early stage, before remodelling and fracture margin resorption occur.^16^

Subtle tarsal and subtarsal pathology can easily be missed in radiography.^17^ The increasing availability of CT and MRI systems for imaging the equine tarsus is allowing earlier detection and more accurate diagnosis of complex tarsal pathology, and standing systems remove the need for general anaesthesia. Cross‐sectional techniques such as CT and MRI are important for definitive diagnosis of CTB fractures, detailed assessment of configuration, and for surgery planning purposes.^2^, ^10^, ^15^, ^18^ Cross‐sectional imaging also allows more detailed detection of other CTB changes such as sclerosis, osseous cyst‐like lesions, demineralisation and osteolysis.^4^, ^5^, ^6^, ^7^, ^8^, ^9^

Variable subchondral bone thickness and sclerosis are common at the distal tarsus and depend on individual activity.^19^ Greater thickness is often observed at proximomedial and distolateral locations. This pattern reflects a shift of compressive loading from medial to lateral at the level of the distal tarsus.^20^, ^21^ Also, subchondral bone thickness at the distal tarsus is altered in horses with tarsal pain. An additional increase in medial subchondral bone thickness may reflect adaptative processes, but whether the abnormal loading pattern is the origin of disease or the result of tarsal pain is unclear.^22^

The aim of this study was to clarify the spectrum and spatial distribution of CTB changes in horses, which are often difficult to detect on radiographs but effectively evaluated with CT scans, as well as to investigate their associations.

## 2 MATERIALS AND METHODS

Computed tomography examinations of equine tarsi performed at the Equine Clinic of the Veterinary Teaching Hospital of the University of Zurich were searched in the PACS system and retrospectively evaluated. Inclusion in the study required availability of diagnostic quality scans of the pelvic limb from the distal aspect of the tibia to the proximal aspect of the metatarsus, including all tarsal structures and regardless of the indication for performing the study. Information regarding the horse's age, weight, breed and use/activity was gathered from electronic patient files and clinical histories.

### 2.1 Image evaluation

**FIGURE 1:**
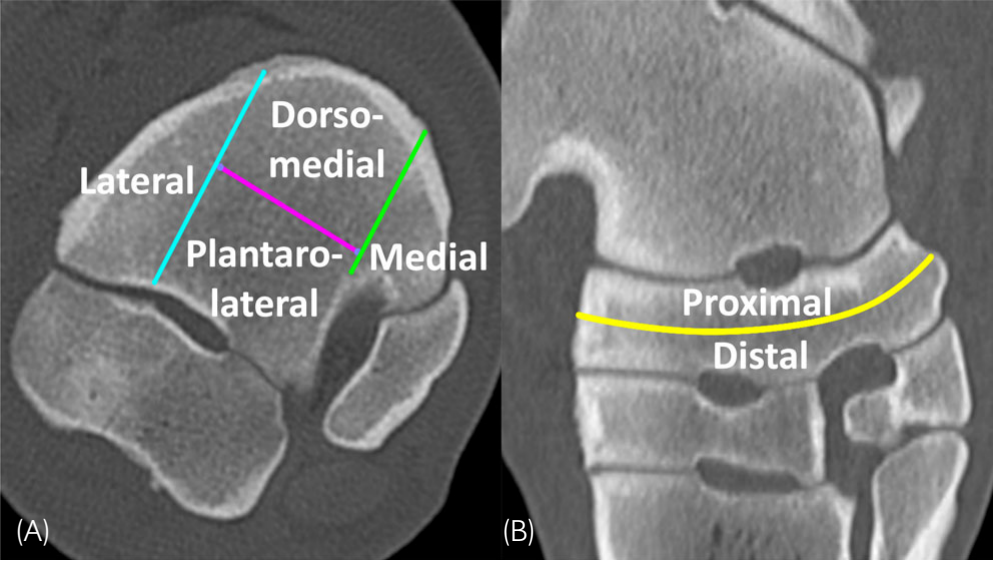
Central tarsal bone subdivision: Transverse image (A) and slightly oblique frontal image (B) depict subdivision of the central tarsal bone. The yellow line represents subdivision in the transverse plane into proximal and distal halves. Light blue and green lines show parasagittal subdivisions in lateral, central and medial thirds. The purple line shows frontal subdivision of the resulting middle third into dorsomedial and plantarolateral regions.

Each CT scan was evaluated by a Diplomate of the European College of Veterinary Diagnostic Imaging and a doctoral student and equine veterinarian with 5 years of equine practice experience. The location(s) of changes were allocated to 8 different regions of the CTB. For this purpose, a subdivision was made along the transverse plane of the bone, halfway through its proximodistal thickness, dividing it into a proximal and a distal half. Each transverse section of the CTB was then divided into four regions. Lines were drawn as shown in Figure 1, based on the centroquartal tarsal joint, one perpendicular to it at the most lateral aspect of the centroquartal intertarsal ligament fossa, one perpendicular to it at the most dorsolateral aspect of the intertarsal ligament fossa between the CTB and the fused first and second tarsal bone, and one parallel to it, through the resulting middle third of the CTB, midway through its dorsoplantar length. In each region, abnormalities or lesions were recorded, including sclerosis, areas of demineralisation, osseous cyst‐like lesions, fissures and fractures. If an abnormality or lesion affected more than one region, its presence was noted in each region.

**FIGURE 2:**
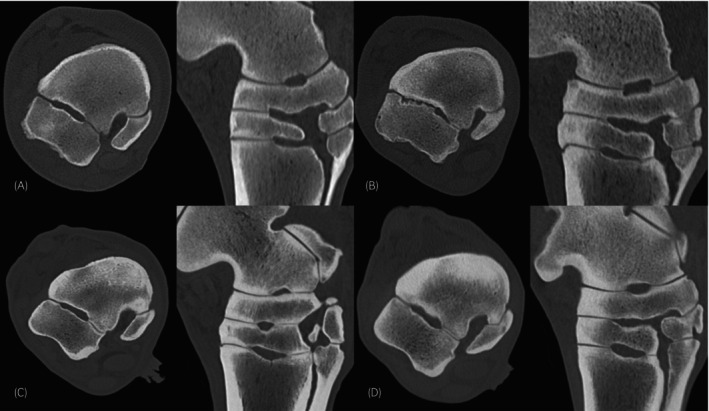
Grading system for sclerosis, based on recognition of normal osseous structure, trabecular pattern and compact‐trabecular distinction. Normal/no sclerosis in (A), characterised by normal osseous structure. Mild sclerosis in (B), characterised by mild increased attenuation of the spongious bone but preserved trabecular pattern and reduced but clear compact‐trabecular distinction. Moderate sclerosis in (C), characterised by moderate increased attenuation of the spongious bone, punctate hypoattenuating inclusions remaining, and moderately reduced compact‐trabecular distinction. Severe sclerosis (D), characterised by homogeneously mineral attenuating regions with complete loss of both trabecular pattern and compact‐trabecular distinction.

**FIGURE 3:**
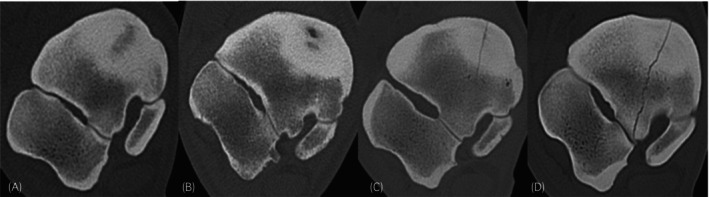
(A) Focal demineralisation in the centre of a sclerotic area. (B) Cyst‐like lesion with soft tissue attenuating centre and sclerotic rim. (C) Fissure of the central tarsal bone in dorsomedial–plantarolateral orientation visible at the dorsomedial aspect and fading into the spongious bone. (D) Complete fracture of the central tarsal bone in dorsomedial–plantarolateral orientation with sclerosis both in the dorsomedial and plantarolateral regions.

Four grades were allocated for sclerosis, based on recognition of typical osseous structure, presence of trabecular pattern, and compact‐trabecular distinction (Figure 2). No sclerosis was assigned when a homogeneous trabecular pattern and clear distinction between compact and trabecular bone were present. Mild increased attenuation of trabecular bone and reduced but clear definition between compact and trabecular bone were graded as mild sclerosis. Moderate sclerosis was assigned when the trabecular pattern was effaced but still recognisable, and compact to trabecular definition was moderately reduced. Severe sclerosis was assigned when the region was homogeneously mineral attenuating and the trabecular pattern was entirely lost. Demineralisation was defined as a focal, ill‐defined area of abnormally reduced, but still mineral attenuation, both in normal and sclerotic bone. An osseous cyst‐like/cystoid lesion was defined as a focal, rounded, well‐defined, soft tissue attenuating region surrounded by a thin, sharply defined sclerotic rim. A fissure was defined as an incomplete linear hypoattenuating discontinuity within the CTB, not extending through compact bone but fading in the trabecular bone. A fracture was defined as a complete linear hypoattenuating discontinuity of the CTB extending through compact bone in all directions. Examples of lesions are shown in Figure 3.

### 2.2 Data analysis

All data were collected in an Excel file. Due to the low number of cases without sclerosis, this variable was reclassified into two categories: no/mild sclerosis and moderate/severe sclerosis. In addition to the variables for each region of the CTB, binary variables were created based on the overall presence or absence of each lesion, regardless of their localisation (demineralisation, cystoid lesion, fissure, fracture).

All analyses were carried out in R Studio (R version 4.3.1). Two‐proportion *Z*‐tests were applied to test for differences between proportions. Chi‐square tests were applied to test for differences between case numbers. Significance levels were set at *p* = 0.05.

## 3 RESULTS

The PACS search yielded 93 tarsal scans in 82 horses, including bilateral tarsal scans in 11 horses. Signalment and CT findings are available in Table S1. Patients included 44 geldings (53.7%), 33 mares (40.2%) and 5 stallions (6.1%), and the mean age was 10.8 years (SD ± 4.8 years, range: 1–26 years). Breeds included 56 warmbloods (68.3%), 7 ponies/Icelandic horses (8.5%), 5 horses each of Iberian (6.1%) and western breeds (6.1%), 3 Freiberger/Haflinger horses (3.7%), 2 Thoroughbreds (2.4%), one horse each of Arab (1.2%), draught (1.2%) and miniature breed (1.2%), as well as one donkey (1.2%). Weight was recorded for 54 patients, and the mean was 521 kg (SD ± 117 kg, range: 140–700 kg). Information about the main activity of the patient was available for 33 cases and included showjumping (*n* = 13, 15.9%), leisure (*n* = 8, 9.8%), dressage (*n* = 5, 6.1%), driving (*n* = 2, 2.4%), western disciplines (*n* = 2, 2.4%), endurance (*n* = 1, 1.2%), eventing (*n* = 1, 1.2%) and racing (*n* = 1, 1.2%). In 19 horses, there was a previous diagnosis of proximal suspensory desmopathy in the examined limb. Seventy tarsal CT scans (75.3%) were performed on recumbent horses, and 23 (24.7%) were performed on standing horses. When the horse underwent both a standing diagnostic CT scan and a recumbent surgery planning/guiding CT scan, only the latter was included, as this was of inherently higher quality due to less patient motion and a smaller scanning field of view. CT examinations included 49 (52.7%) scans of the right pelvic limb and 44 (47.3%) scans of the left pelvic limb. The CTB was identified as the primarily relevant site of pathology in 25 tarsi, and details of diagnostic analgesia were available in 14/25. These included four positive tarsometatarsal joint blocks, three positive tarsocrural joint blocks, three positive blocks proximal to the tarsus, two positive high 4‐point blocks and two positive plantar lateral nerve blocks.

### 3.1 Sclerosis

A total of 98.4% of examined regions (366/372) showed mild or higher degrees of sclerosis at least at one location. The medial aspect of the CTB was free of sclerosis in 4 (4.3%), affected by mild sclerosis in 69 (74.2%) cases, by moderate sclerosis in 13 (14.0%) and by severe sclerosis in 7 (7.5%). The dorsomedial aspect of the CTB was free of sclerosis in 1 case (1.1%), affected by mild sclerosis in 8 (8.6%) cases, by moderate sclerosis in 54 (58.1%) and by severe sclerosis in 30 (32.3%). The plantarolateral aspect of the CTB was free of sclerosis in 1 case (1.1%), affected by mild sclerosis in 75 (80.6%) cases, by moderate sclerosis in 12 (12.9%) and by severe sclerosis in 5 (5.4%). The lateral aspect of the CTB showed sclerosis in all tarsi, mild in 24 (25.8%) cases, moderate in 61 (65.6%) and severe in 8 (8.6%).

### 3.2 Demineralisation

An area of demineralisation was present in 32/372 (8.6%) examined regions. One tarsus showed demineralisation at the distal subchondral bone medially. Twenty‐four tarsi showed demineralisation dorsomedially, 21 at the proximal subchondral bone, 2 at the distal subchondral bone and one both at the proximal and the distal subchondral bone. Five tarsi showed demineralisation at the plantarolateral aspect, one at the proximal subchondral bone, and four at the distal subchondral bone. Two tarsi showed demineralisation at the lateral aspect, one at the proximal subchondral bone, and one at the distal subchondral bone.

### 3.3 Cystoid lesions

A cystoid lesion was present in 27/372 (7.3%) examined regions. Thirteen cystoid lesions were located medially, all at the distal subchondral bone. Eight cystoid lesions were located dorsomedially, 5 at the proximal and 3 at the distal subchondral bone. In the plantarolateral region, there were five cystoid lesions, one at the proximal subchondral bone and four at the distal subchondral bone. In the lateral region, there was one cystoid lesion located at the distal subchondral bone.

### 3.4 Fissures/fractures

Fissures or fractures of the CTB were present in 24/93 (25.8%) examined tarsi and in 43/372 (11.6%) examined regions. The medial region of the CTB displayed a fracture in two tarsi. Fissures and fractures were present respectively in 7 and 15 dorsomedial regions. In the plantarolateral aspect, there were 4 fissures and 12 fractures. In the lateral aspect, there were three fractures. Seven of seven fissures and 12/17 fractures were in a DMPL direction. The remaining five fractures showed a variable configuration.

### 3.5 Associations

The proportion of tarsi with moderate/severe sclerosis in the dorsomedial region (90%) was significantly higher than the proportion of cases with moderate/severe sclerosis in the other regions (lateral 74%, *Z*‐statistic: 2.9, *p* = 0.004; medial 22%, *Z*‐statistic: 9.5, *p* < 0.001; plantarolateral 18%, *Z*‐statistic: 11.4, *p* < 0.001). The proportion of tarsi showing lesions dorsomedially was significantly higher in tarsi with moderate/severe sclerosis dorsomedially (35 out of 84), compared to tarsi showing no/mild sclerosis dorsomedially (0 out of 9) (chi‐square: 4.4, *p* = 0.04). The proportion of tarsi showing lesions in the plantarolateral region was higher in tarsi with moderate/severe sclerosis plantarolaterally (10 out of 17) than in tarsi with no/mild sclerosis in this region (13 out of 76) (chi‐square: 10.8, *p* = 0.001). The proportion of tarsi showing demineralisation in the plantarolateral region was higher in the presence of moderate/severe sclerosis (4 out of 17) than in cases with no/mild sclerosis in this region (1 out of 76) (chi‐square: 9.5, *p* = 0.002). The proportion of tarsi showing fissures in the plantarolateral region was higher in cases with moderate/severe sclerosis (3 out of 17) than in cases with no/mild sclerosis in this region (1 out of 76) (chi‐square: 5.5, *p* = 0.02). Of 24 tarsi with fissures or fractures, 17 showed concurrent demineralisation. This is significantly higher than in tarsi without fissures or fractures (69), of which only 11 showed demineralisation (chi‐square 23.0, *p* < 0.001). The prevalence of lesions, regardless of location, was significantly associated with higher degrees of dorsomedial sclerosis, as the proportion of tarsi that showed lesions (demineralisation, cystoid, fissure, fracture) in any region of the tarsus was higher in tarsi with moderate/severe sclerosis dorsomedially (57%, 48 out of 84) than in tarsi with no/mild sclerosis dorsomedially (0 out of 9) (chi‐square: 8.5, *p* = 0.004). The proportion of tarsi with lesions in any region was higher with moderate/severe plantarolateral sclerosis (82%, 14 out of 17) than with no/mild plantarolateral sclerosis (45%, 34 out of 76) (chi‐square: 6.4, *p* = 0.01). Of five fissure/fractures with variable, non‐DMPLO configuration, none showed associated demineralisation.

## 4 DISCUSSION

By retrospective analysis of all tarsal CT scans performed at our institution, we identified a multitude of CTB changes with consistent patterns and predilection sites for changes. As already identified by other studies, CTB sclerosis was very common in our population. By applying our grading system, most evaluated CTBs showed some degree of sclerosis in at least one of the assessed regions. The slightest increase in attenuation of the spongious bone was classified as mild sclerosis. This might be inadequate to evaluate clinical cases and should not be directly interpreted as pathological or clinically significant. Some degree of CTB sclerosis, just as at other predilection sites, likely reflects normal osseous adaptation to stress and loading, which in turn is dependent on conformation, age, discipline and intensity of training, among other factors. When combining cases with no and mild sclerosis as one group, 85 of 93 tarsi examined displayed at least one area with moderate or severe sclerosis. Additionally, we found sclerosis to be significantly more common and of significantly higher degree in the dorsomedial region of the CTB, which raises the suspicion that this region is the area of main subchondral bone stress/loading. Previous studies have identified areas of increased stress of the CTB, which were explained to reflect a shift of compressive load from medial to lateral at the level of the distal tarsal joints.^21^ The question of whether mild sclerosis can be normal and a high degree necessarily means pathology and clinical impact cannot be answered in this study. Modalities identifying bone fluid or increased osseous metabolism, such as MRI and scintigraphy/PET might help answer this question.

The distribution of fissures and fractures was highly consistent, often affecting the dorsomedial and plantarolateral areas in our study and agreeing with the previously identified DMPL direction of fractures described in other studies.^7^, ^8^ We additionally showed a significant association between fissures/fractures and higher degrees of sclerosis, supporting a stress‐related aetiology.

**FIGURE 4:**
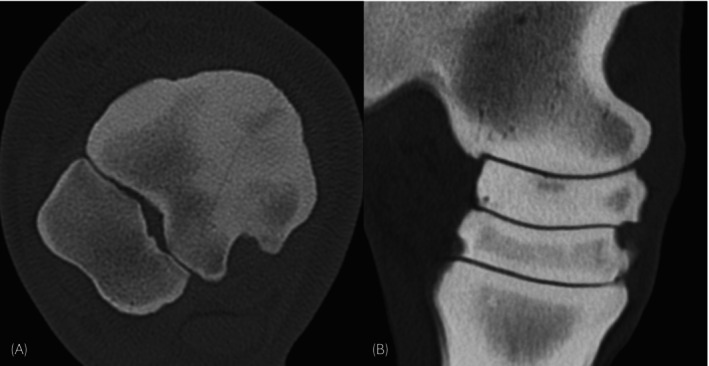
Examples of combination of central tarsal bone (CTB) changes. (A) Transverse image showing marked sclerosis extending through most of the dorsomedial–plantarolateral length of the CTB, together with a focal area of dorsomedial demineralisation and a fracture line coursing through it. (B) Slightly oblique frontal image, with a focal area of demineralisation visible at the proximodorsomedial aspect of the CTB without evident fissure or fracture.

A previously underestimated observation was the presence of demineralisation at the proximal aspect of the dorsomedial region of the CTB, in the centre of the often‐encountered sclerotic area. Also, the majority of fissures and fractures were associated with or coursed through these areas of demineralisation at the proximal aspect of the dorsomedial region of the CTB (Figures 4 and S1). The association of fissures and fractures with a diffuse area of sclerosis and a focal area of demineralisation additionally supports a stress‐related aetiology, as this has been shown to be present at other more commonly described fracture sites.^23^, ^24^ We speculate that this type of CTB demineralisation might represent a focus of subchondral bone failure in the centre of sclerosis, leading to a weak spot and resulting fissure or might indicate margin resorption at an existing fissure, primarily developing in less elastic sclerotic bone. Observations from our study, with horses displaying demineralising foci in sclerotic bone without evidence of fissures and our finding that most DMPL fissure/fractures presented with a surrounding demineralising area, favour the former hypothesis. Thus, we postulate that proximodorsomedial sclerosis of the CTB and a central demineralisation represent prodromal findings. Nevertheless, a combination or continuum of overlapping osseous remodelling processes is likely present. In contrast to this observation, fractures with other configurations, not in a DMPL direction, were not associated with demineralisation. Subchondral bone damage can be clinically relevant and cause relevant lameness in the absence or before the development of fissures and fractures, as shown at other locations of subchondral bone stress, such as in POD or sagittal proximal P1 fractures, respectively. Therefore, we tend to extrapolate that similar lesions in the CTB may be clinically relevant prodromal signs of fracture. Bone metabolism imaging modalities such as scintigraphy or PET could help determine the activity and clinical relevance of a CTB abnormality, such as moderate/severe sclerosis, demineralisation or cyst‐like lesions.

**FIGURE 5:**
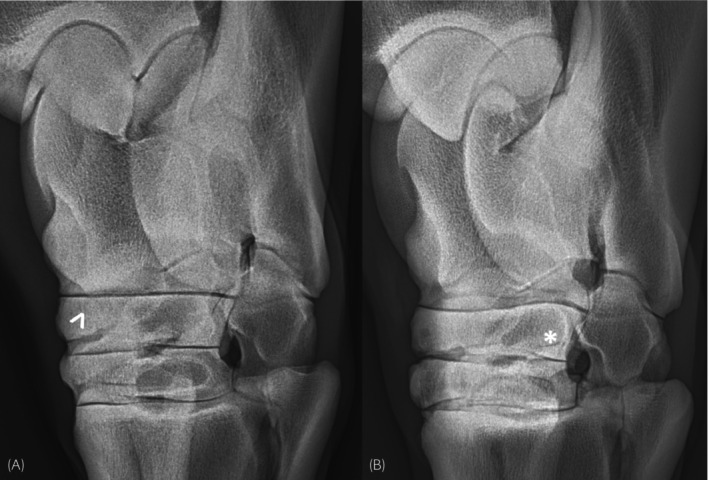
Examples of tarsal radiographs which should be carefully scrutinised for subtle changes when central tarsal bone pathology is suspected. In (A), a focal ill‐defined lucent area (white arrowhead) is visible in the centre of a larger region of sclerosis at the proximodorsomedial aspect of the central tarsal bone. In (B), an area of moderate sclerosis is visible at the plantarolateral aspect of the central tarsal bone (asterisk). In both these horses, computed tomography revealed a fracture of the central tarsal bone in dorsomedial–plantarolateral orientation, which was not previously identified in radiography.

An additional important observation with practical clinical relevance is that dorsomedial sclerosis, which extends to affect the plantarolateral area of the CTB, was significantly associated with lesions such as demineralisation, fissures and fractures. As CT might not be readily available to diagnose CTB lesions in detail, and some lesions might only be visible when projected precisely tangential to the x‐ray beam, this is of particular interest in practice as the presence of sclerosis visible in the plantarolateral half of the CTB on a DLPMO projection should alert for the presence of additional lesions (Figure 5).

In contrast to sclerosis, demineralisation, fissure and fractures, a more random distribution was observed for osseous cyst‐like lesions, which were often identified at the distal aspect of the medial region of the CTB. Therefore, a different aetiology has to be postulated, either unrelated to subchondral bone stress or related to other types of stress. Subchondral osseous cyst‐like lesions have been described at sites of increased subchondral bone stress,^25^ but the reason for subchondral bone to develop a cyst rather than a fissure is unclear. An association with a different type of loading pattern, possibly more focal than linear, or association with individual CTB shape and conformation can only be hypothesised without further research.

This study has multiple limitations. First, it is affected by limitations inherent to its retrospective design, where standardised case details and imaging protocols were unavailable, leading to missing information and variability in image quality. As the findings are purely correlative, causative explanations for the associations between the observed changes are speculative and warrant experimental confirmation. Also, the described findings, discussed hypotheses and postulations might only apply to a heavily warmblood‐oriented population engaging in English disciplines, particularly showjumping, dressage and eventing. We sporadically encountered difficulties in assessing the degree of sclerosis in CTs performed on standing horses, as motion artefacts limit the interpretation of fine trabecular bone structure, possibly leading to erroneously higher degrees of sclerosis assigned. Agreement was achieved between observers by reviewing these cases together, based on previously stated definitions of sclerosis degrees.

Another limitation was the chosen subdivision of the CTB in unevenly sized regions, which might be responsible for a bias, resulting in more changes and lesions present in the larger areas, particularly dorsomedially and plantarolaterally. A more evenly sized subdivision might have mitigated this, but defining regions based on anatomic landmarks was preferred for a more standardised subdivision across all tarsi.

In isolated cases, we had difficulties differentiating between osseous cyst‐like lesions and a focal area of demineralisation, particularly when the image was affected by motion artefacts. Distinction between focal demineralisation, degenerative or maladaptive cystoid lesions, and developmental osseous cyst‐like lesions can be difficult. A spectrum of pathology is plausible for non‐developmental cystic lesions, but studies are lacking in horses. Irregularly marginated, ill‐defined areas of hypo‐ but still mineral attenuation possibly represent more recent or immature processes. In contrast, a rounded, well‐defined, thin‐walled soft tissue attenuating lesion not surrounded by sclerosis could represent the chronic end‐stage remodelled inactive scar.

Little information was available in the patients' records that could help derive clinical relevance of the described CT changes in our population. Most of the severe lesions, such as fissures and fractures, were easily identified as responsible for the presenting signs. In cases of mild changes or sclerosis only, which can represent normal stress adaptation of bone substance to stress, it is unclear if these findings were the cause of lameness. Also, when information was available, marked heterogeneity was present in the pattern of positive diagnostic anaesthesia. Distal tarsal blocks, which were often positive in our cases of CTB pathology, overlap with other common sites of pathology, such as the tarsometatarsal joint and suspensory ligament origin. Additional studies, including precise details of regional anaesthesia and utilising advanced imaging to display bone composition and metabolism, are required to investigate the association between CTB changes and clinical signs. In particular, the clinical relevance of sclerosis alone and smaller lesions, such as mild demineralisation, requires future clarification.

In conclusion, sclerosis of the CTB is a common finding of questionable clinical relevance if found alone, but shows consistency in its distribution, particularly prevalent at the proximodorsomedial aspect. Lesions of the CTB often occur in combination with higher degrees of sclerosis and fissures/fractures were associated with demineralisation. This combination of findings suggests similarities with other more recognised areas of subchondral bone injury, resulting in fractures. Dorsomedial demineralisation and sclerosis extending plantarolaterally should be carefully evaluated in tarsal imaging as they could be associated with fissure/fractures or represent a prodromal change.

## FUNDING INFORMATION

None.

## CONFLICT OF INTEREST STATEMENT

The authors declare no conflicts of interest.

## AUTHOR CONTRIBUTIONS

**Sandra Campana:** Investigation; writing – original draft; writing – review and editing; data curation; methodology. **Marie Dittmann:** Conceptualization; methodology; data curation; formal analysis. **Patrick Kircher:** Supervision; conceptualization; writing – review and editing. **Brice Donati:** Project administration; conceptualization; investigation; writing – original draft; validation; visualization; writing – review and editing; supervision; resources; data curation.

## DATA INTEGRITY STATEMENT

Brice Donati had full access to all the data in the study and takes responsibility for the integrity of the data and the accuracy of the data analysis.

## ETHICAL ANIMAL RESEARCH

Research ethics committee oversight not required by this journal: retrospective study of clinical records.

## INFORMED CONSENT

Explicit owner consent for inclusion of animals in this study was not obtained. Owners/trainers were made aware that case information may be used for research in general.

## ANTIMICROBIAL STEWARDSHIP POLICY

Not applicable.

## Supporting information


**Table S1.** Signalment and CT findings associated with the central tarsal bone in 93 limbs.

**Figure S1.** Transverse computed tomography images of 15 cases with central tarsal bone fracture or fissures, showing a consistent pattern but variable degrees of surrounding dorsomedial sclerosis and demineralisation.

## Data Availability

The data that support the findings of this study are openly available in ZORA at https://www.zora.uzh.ch/id/eprint/279459/, doi:10.5167/uzh-279459, reference number 279459.
