# Supplementary material for: Computed tomography identifies the proximodorsomedial subchondral bone of equine central tarsal bones as a predilection site for sclerosis, demineralisation and associated fractures
Source: Equine Vet J. 2025 Jul 24;58(3):797–804. doi: 10.1111/evj.70001 (PMC13041607; doi:10.1111/evj.70001)

Equine Veterinary Journal BEVA

Figure S1: Transverse computed tomography images of 15 cases with central tarsal bone fracture or fissures, showing a consistent pattern but variable degree of surrounding dorsomedial sclerosis and demineralisation.

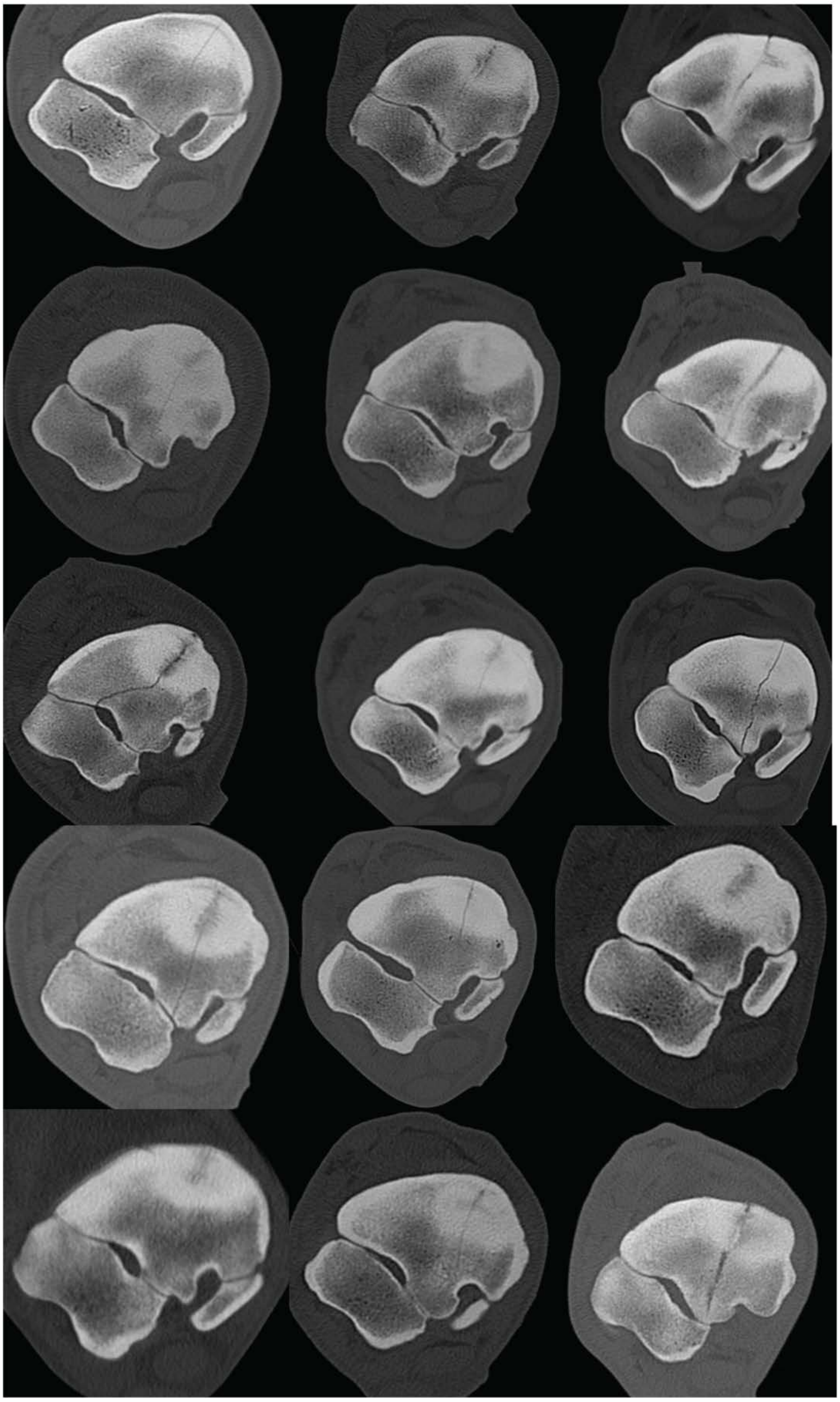

Supplement: Supplementary file 1 — Table S1. Signalment and CT findings associated with the central tarsal bone in 93 limbs. [file EVJ-58-797-s001.pdf]
